# Supplementary material for: Layer-by-Layer Heterostructure of MnO2@Reduced Graphene Oxide Composites as High-Performance Electrodes for Supercapacitors
Source: Membranes (Basel). 2022 Oct 26;12(11):1044. doi: 10.3390/membranes12111044 (PMC9697611; doi:10.3390/membranes12111044)
Supplement: Supplementary file 1 [file membranes-12-01044-s001.zip › membranes-1952956-supplementary.pdf]

Supplementary Information

Layer−by−Layer Heterostructure of MnO_2@Reduced Graphene Oxide Composites as High−Performance Electrodes for Supercapacitors

Tingting Liu [1, 3,] *, Lei Chen [1], Ling Chen [2,] *, Guoxing Tian [2], Mingtong Ji [1, 3], Shuai Zhou [2]

[1] Qinhuangdao Key Laboratory of Marine Oil and Gas Resource Exploitation and Pollution Prevention, Northeast Petroleum University at Qinhuangdao, Qinhuangdao 066004, P. R. China

[2] Hebei Key Laboratory of Applied Chemistry, College of Environmental and Chemical Engineering, Yanshan University, Qinhuangdao 066004, P. R. China

[3] Provincial Key Laboratory of Polyolefin New Materials, College of Chemistry & Chemical Engineering, Northeast Petroleum University, Daqing 163318, P. R. China

*Corresponding authors:

E−mail: 2008little@163.com (T. Liu), hhchen@ysu.edu.cn (L. Chen).

Preparation of GH anode materials

50 mg of GO was dispersed in deionized water (25 mL) under ultrasound for 1 h, followed by stirring for 1h. The solution was transferred into Teflon−lined stainless steel autoclave (50 mL) and reacted at 90 °C for 3 h to obtain graphene hydrogel (GH).

Preparation of samples for FTIR

The sample (~1 mg) and dry KBr (S. P., ~200 mg) were mixed uniformly in an agate mortar. After fully grinding, the mixture was transferred into the mold evenly. Then the mold was put into the tablet press and kept under the pressure of 20 MPa for 1−2 minutes to get a thin wafer.

Table S1. Zeta potential of e−MnO_2 regulated by different concentrations of PDDA

Sample	e−MnO_2−0.5	e−MnO_2−0.75	e−MnO_2−1
Concentration of PDDA (g/L)	0.5	0.75	1
Zeta potential (mV)	27.0	35.7	30.2

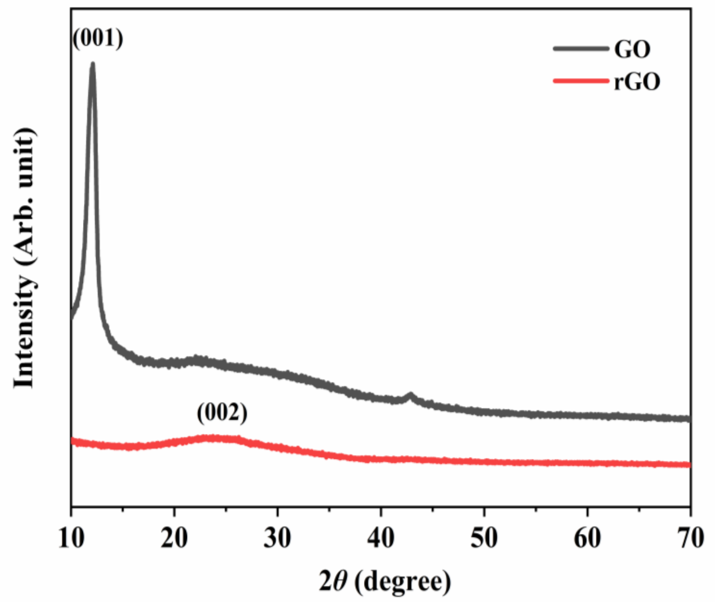

Figure S1. XRD patterns of GO and rGO.

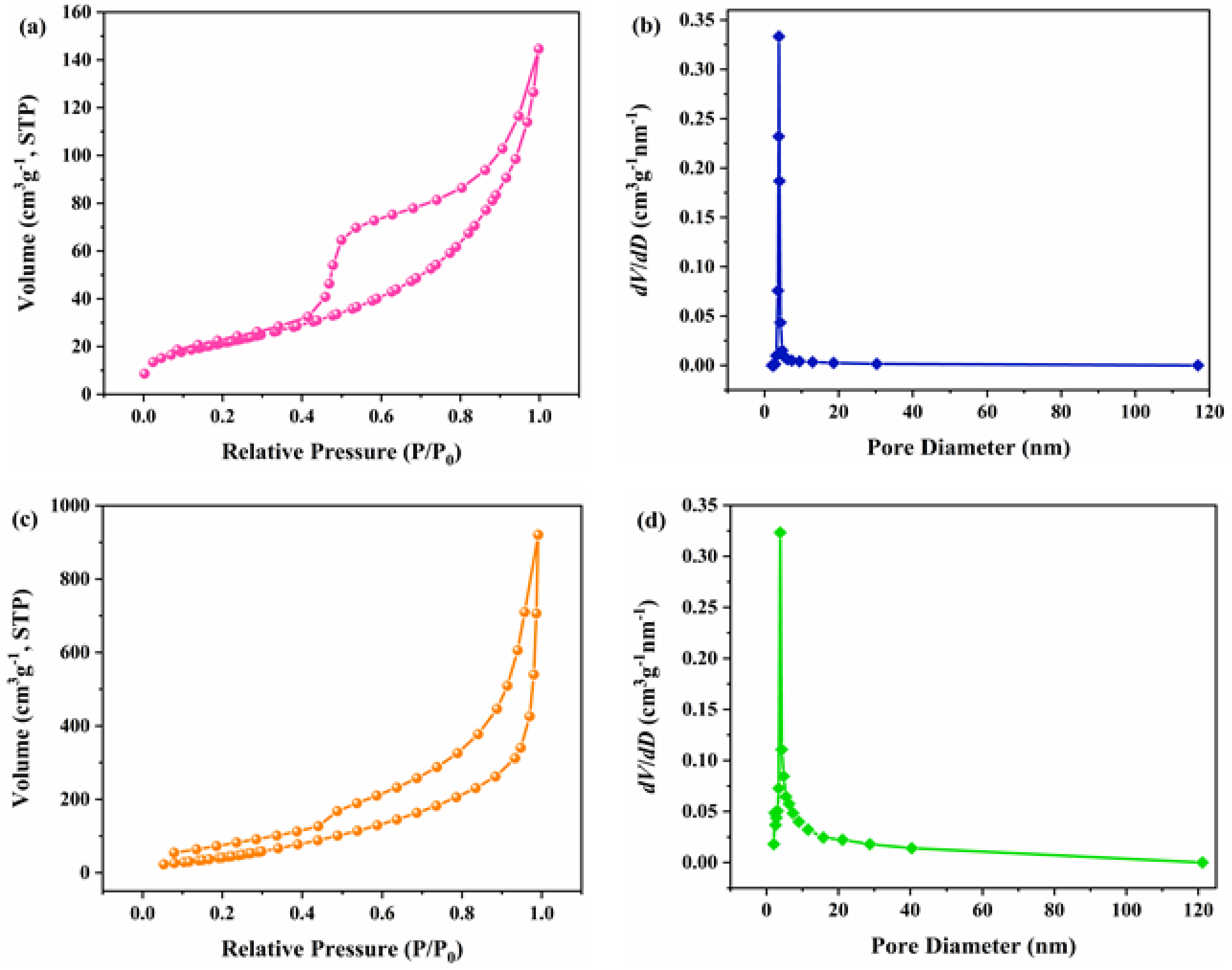

Figure S2. Adsorption−desorption isotherms of **(a)** MnO_2 and **(c)** rGO and the pore−size distribution of **(b)** MnO_2 and **(d)** rGO.

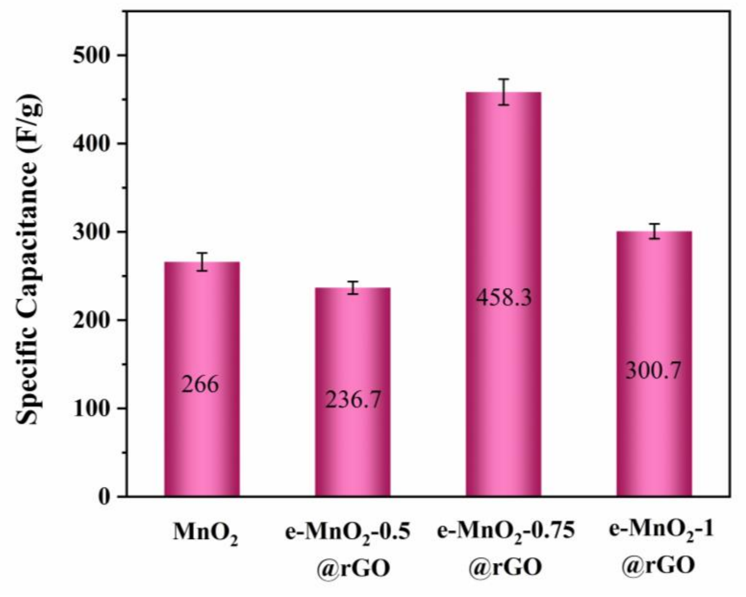

Figure S3. The average specific capaitance of MnO_2, e−MnO_2−0.5@rGO, e−MnO_2−0.75@rGO and e−MnO_2−1@rGO (1 A/g). Error bars represent standard deviations from measurements of three different samples.
